# SLNs and NLCs for Skin Applications: Enhancing the Bioavailability of Natural Bioactives

**DOI:** 10.3390/pharmaceutics16101270

**Published:** 2024-09-28

**Authors:** Diana Antonia Safta, Cătălina Bogdan, Mirela-Liliana Moldovan

**Affiliations:** Department of Dermopharmacy and Cosmetics, Faculty of Pharmacy, “Iuliu Hatieganu” University of Medicine and Pharmacy, 12 I. Creanga Street, 400010 Cluj-Napoca, Romania; diana.an.safta@elearn.umfcluj.ro (D.A.S.); mmoldovan@umfcluj.ro (M.-L.M.)

**Keywords:** solid lipid nanoparticles, nanostructured lipid carriers, herbal extracts, essential oils, phytocompounds

## Abstract

Natural bioactives are mixtures of compounds extracted from plants with physicochemical properties that are usually not favorable for penetrating the skin’s complex barrier. Nanoparticles have important advantages both in dermatology and cosmetology: improved solubility and stability of encapsulated phytocompounds, controlled and sustained skin delivery, and enhanced skin permeation, leading to an improved bioavailability. This review focuses on two generations of lipid-based nanoparticles: solid lipid nanoparticles (SLNs) and nanostructured lipid carriers (NLCs). An extensive overview on the recent studies on SLNs and NLCs entrapping essential oils, oils, herbal extracts, and phytocompounds for topical applications is presented, emphasizing their composition, physicochemical characterization, efficacy, and methodologies used to evaluate them. This review also summarizes topical systems containing natural bioactives incorporated into SLNs and NLCs, commercially available products and registered patents in the field. SLNs and NLCs turn out to be effective nanocarriers for skin applications, offering significantly improved encapsulation efficiency, stability, and bioactives delivery. However, their full potential is underexplored. Future applications should study the encapsulation potential of new natural bioactives and show more specialized solutions that address specific requirements; an improved product performance and a pleasant sensory profile could lead to increased customer compliance with the product use.

## 1. Introduction

The entrapment of herbal extracts (HEs), essential oils (EOs), or phytocompounds (PCs) in novel pharmaceutical nanostructures for skincare represents a blend of traditional knowledge with modern technology, aligning with the growing consumers’ preference for natural products. Their effectiveness and safety, along with the current trend towards eco-friendly products, encourage this nature-inspired approach. Thus, according to the World Health Organization (WHO), over 80% of people worldwide obtain their primary healthcare from traditional medicine, based on natural-origin products [1,2].

HEs and EOs are rich in bioactive PCs such as polyphenols, flavonoids, and terpenoids, which have been shown to possess potent antioxidant, anti-inflammatory, and antimicrobial properties. Thus, as they can offer a wide range of important effects and can be useful to various skin types and conditions, they are highly versatile in cosmetic and dermatological formulations. Nanotechnology can help unlock the full potential of traditional herbal extracts by improving their stability, bioavailability, and delivery, expanding their use in modern dermatology and cosmetology [3].

## 2. Skin Delivery of SLNs and NLCs

The skin, as the external covering of the body, has multiples roles, with the primary function being to protect it against environmental factors and maintain body homeostasis. For many years, the skin was considered as an impenetrable barrier; however, nowadays it is known that skin is permeable to various substances, depending mainly on their physicochemical properties and on the skin integrity. Due to its selective permeability, the skin can also be considered a therapeutic interface and a target for drug delivery at three levels: on the skin surface (e.g., for antiseptic effect, repellent effect against insects, skin protection effect), at the epidermis and dermis levels (e.g., for anti-inflammatory, antimicrobial, antineoplastic effects), and for transdermal delivery when the drug is expected to reach systemic circulation to provide a systemic effect, representing an alternative to oral administration [4,5].

### 2.1. Pathways of Skin Absorption

The penetration of actives through intact skin is obtained through two pathways: transepidermal penetration and transappendageal penetration (via sweat glands, sebaceous glands, and hair follicles). In transepidermal penetration, the stratum corneum governs the penetration of active ingredients either through intracellular or intercellular pathways, acting as a barrier for molecules with high molecular weight (MW) and hydrophilic molecules [6,7]. Skin absorption involves three successive phases: the penetration phase, consisting of a passive diffusion into the *stratum corneum* of the active ingredient from the topical product applied to the skin surface, followed by a permeation phase where the active ingredient passes through the viable epidermis to dermis, and the resorption phase, when the active ingredient passes from the dermis into the skin’s microcirculation and subsequently into deeper skin layers or into systemic circulation [8].

### 2.2. Skin Permeability of Active Ingredients

The penetration across the skin is influenced by several factors like the physiological properties of the skin, the physicochemical properties of the incorporated active ingredient, and the properties of the vehicle. Regarding skin properties, one of the most important features is the quality of *stratum corneum* (its integrity, thickness, and hydration state), along with the skin temperature, which has a direct influence on skin microcirculation. Other factors such as age, gender, anatomical site, and application method (e.g., massage) are also mentioned [9].

Among the physicochemical properties of the actives, the skin penetration may be increased when the active’s solubility is increased, either by reducing particle size or by obtaining supersaturated vehicles. According to Bos et al., small molecules with an MW under 500 Dalton can pass intracellularly, while lipophilic actives can pass into skin via intercellular lipids, following the intercellular route. The penetration of actives into the skin is largely dependent also by the lipid–water partition coefficient, as molecules with strong hydrophilic or lipophilic character tend to have small permeation across the skin, while a good solubility in aqueous and lipophilic environments will ensure a good penetration ability. Usually, a value of the octanol–water partition coefficient of 1–3 will ensure a good permeability through skin. Additionally, the melting point of the active should be below 200 °C to facilitate its skin permeation [7,10,11,12,13].

The vehicle used to deliver the actives impacts skin penetration by ensuring good skin contact and providing occlusion. For some active ingredients, the pH of the vehicle is important to facilitate the active permeation through the skin, as it influences the ionization state of the active; thus, its permeation across *stratum corneum* lipids is influenced. Knowing that only the unionized form of an active ingredient can permeate the skin, for an active in unionized form, the diffusion coefficient may be higher [14]. Occlusion usually reduces transepidermal water loss (TEWL) and increases skin hydration by up to 50%, leading to swollen corneocytes and facilitating water retention in intercellular lipids. This can affect the skin barrier and may increase the permeability of lipophilic active ingredients, while for hydrophilic active ingredients the influence of occlusion on skin permeation is less important [15]. Histological changes such as the expanding of the intercellular space of the stratum spinosum were recorded at 6 h of occlusion. After 24 h of occlusion, additional changes include the appearance of perinuclear vacuoles in the keratinocytes (excluding the stratum granulosum) and the presence of vesicles and vacuoles in the cytoplasm in the cellular periphery. Microbiological modifications in skin microbiota were also noticed in occluded skin, usually the increase in bacterial colonies, namely, *Staphyococcus* sp., *Corynebacterium* sp., and of the yeast *Pityrosporum orbiculare* [16]. The occlusion effect of topical products may be modulated by choosing the right ingredients and the galenic formulation. Vehicles with different degrees of occlusion may be obtained when using mineral and vegetal oils, paraffin, waxes, and other fats. Regarding the galenical formulations, the ointments are the most occlusive, followed by water-in-oil emulsions, while the oil-in-water emulsions are less occlusive [17]. Vehicles may also influence skin permeation through specific ingredients known as permeation enhancers. There are well-known substances like alcohols (ethanol, butanol, propanol, decanol, lauryl alcohol), esters (glyceryl monocaprylate, glyceryl monooleate), and surfactants, which enhance skin permeation of polar and nonpolar actives by lipid extraction; ethanol enhances skin permeation also by fluidization of intercellular lipid matrix, soap-like detergents enhance skin permeation through the disorganization of the *stratum corneum* lipid lamellae; other ingredients like proteins, lipids, and surfactants act by disintegration of the tight junctions structure [18,19]. Other chemical penetration enhancers belong to ethers (Diethylene glycol monoethyl ether or Transcutol^®^), terpenes (D-limonene, menthol), sesquiterpenes (farnesol, neridol), glycols (propylene glycol, dipropylene glycol), sulfoxides (dimethyl sulfoxides, decyl methyl sulfoxides), amides (azone), carboxylic acids and esters (oleic acid, geranic acid, caprylic acid, lauric acid) pyrrolidone derivatives (N-methyl-2-pyrrolydone), and amino acids derivatives [18]. Other ingredients like pyrrolidone carboxylic acid, proline, and 4-hydroxyproline act like transdermal permeation enhancers, but with reversible effect [20]. Some of these permeation enhancers may be used in the composition of the SLNs and NLCs.

## 3. Fundamental Insights into SLNs and NLCs

**Figure 1:**
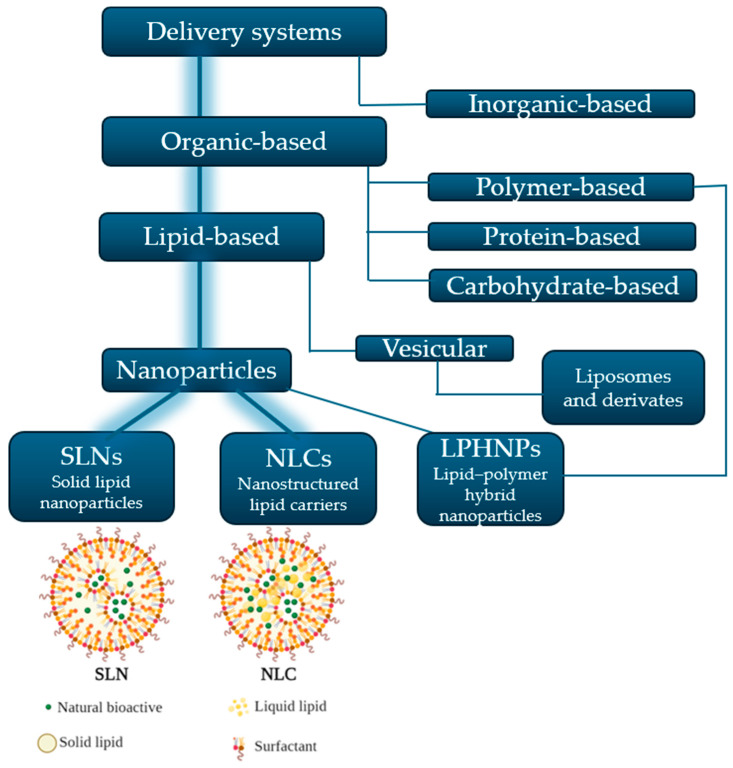
The classification of delivery systems, emphasizing the subcategories of lipid-based nanoparticles (image created using Biorender.com, accessed on 16 September 2024).

Nanosystems have emerged as revolutionary platforms for encapsulating both synthetic and natural bioactives (HEs, EOs, PCs), enhancing their efficacy and bioavailability [21]. During the last decades, a wide range of inorganic- and organic-based delivery systems were developed, with their classification being presented as a schematic diagram in Figure 1. From the organic delivery systems, the lipid-based ones are considered the most appropriate systems for topical applications due to their structure, biocompatibility, and resemblance to skin components [22,23]. Lipid-based nanoparticles consist of vesicular systems (liposomes and derivates), lipid nanoparticles—SLNs, NLCs, and the hybrids between lipid- and polymer-based nanoparticles, lipid–polymer hybrid nanoparticles (LPHNPs), respectively [24,25]. SLNs and NLCs loading natural bioactives, according to their lipophilicity, are also represented in Figure 1, emphasizing their main structural components.

SLNs and NLCs are considered a better alternative than vesicular systems because they can penetrate the deeper layers of the epidermis and can ensure higher stability and prolonged and sustained release of the incorporated actives [1,25]. Moreover, while liposomes have been extensively studied so far to incorporate HEs or PCs, lipid nanoparticles are more recently used for this purpose and, therefore, this topic is less covered. SLNs and NLCs can entrap various types of active ingredients, both lipophilic and hydrophilic, within their lipid matrix [1]. According to their lipophilicity, the active ingredients are dissolved or molecularly dispersed in the solid lipid matrix (Figure 1), restricting their mobility, thus providing a controlled and sustained release profile [26].

SLNs and NLCs are typically composed of solid and/or liquid lipids (0.1–30%), water, and surfactants or emulsifiers (0.5–5% *w*/*w*), as represented in Figure 1 [26]. They have the major advantage of being safer than polymeric nanoparticles both for the skin and for the environment, since organic solvents are not used during the synthesis phase [1,25], leading to a natural and sustainable fabrication [27].

SLNs consist of solid lipids and surfactants (Figure 1). They are solid colloidal particles both at room and body temperature and have better target capability, lower toxicity due to the presence of physiological lipids, enhanced bioavailability, and better ability to protect drugs or natural bioactives [1]. SLNs have good physical and chemical stability that makes them a preferable alternative for emulsions and liposomes [28]. Since their development, at the beginning of the 1990s, parenteral, oral, and ocular delivery of SLNs have been the subject of extended research, proving better bioavailability of entrapped active ingredients. SLNs are regarded as promising carriers for topical application also, due to their capacity to improve drug penetration into the skin. Particularly, they are well suited for usage in injured or inflamed skin, because the lipids used to prepare SLNs are nontoxic and nonirritating [29]. However, SLNs may have some disadvantages such as instability during storage, potential particle aggregation or low encapsulation efficiency for some natural bioactives [30].

NLCs are considered the second generation of SLNs, due to their better encapsulation efficiencies and improved stability. NLCs are formed by surfactants and solid and liquid lipids (Figure 1), but their aggregate state also remains solid at the room and body temperature [1]. The lipids from NLCs have a less ordered crystalline arrangement than SLNs, which is useful to prevent the leakage of the entrapped active ingredients [1,25]. The structural differences between SLNs and NLCs could be explained considering the key role of the oil phase (liquid lipid) allowing them to retain the active ingredients inside the NLC matrix and to release them in a controlled manner. The inclusion of oils is useful to hold higher amounts of an active ingredient because the oils, behaving as an impurity in the solid lipids, reduce the particle crystallinity, ensuring better stability and a higher suitability for a controlled release [31]. Moreover, San et al. mentioned that NLCs with a higher ratio of liquid lipids led to a better physical stability. Conversely, NLCs with lower liquid lipid ratio may have lower physical stability; however, they can show potential for topical application due to their better skin absorption [32].

SLNs and NLCs can be used to entrap active ingredients by encapsulation, incorporation, or/and surface attachment, and to deliver them to target tissues [1]. Depending on the preparation method and the type of loading of active ingredients in the lipidic network, three types of SLNs and NLCs can be distinguished, as summarized in Table 1, together with their key features [30,33].

Small changes in formulation can influence SLNs and NLCs structure and, consequently, their applicability. To achieve a prolonged drug release, for instance, SLNs type III is adequate, while SLNs type II is not appropriate for this purpose. In contrast, SLNs type I can exhibit controlled release qualities [30,33,34].

SLNs and NLCs are primarily intended to deliver lipophilic active ingredients, whereas the vesicular carriers are primarily intended to deliver hydrophilic active ingredients. Particularly, SLNs have a greater affinity for lipophilic active ingredients than for hydrophilic ones because their fundamental matrix is solid lipid-based. Because of this, it has been observed that SLNs containing hydrophilic active ingredients exhibit limited stability or low entrapment efficiency. During the preparation process, hydrophilic active ingredients have a high tendency to partition into the outer aqueous phase because they lack affinity for the lipid matrix but, hydrophilic drug-loaded SLNs with good entrapment efficiency may be obtained as well, even if it is considered a challenging objective [35].

The HEs often contain a mixture of both hydrophilic (phenolic acids, flavonoids, glycosides, some alkaloids) and lipophilic compounds (terpenoids, essential oils, fatty acids, steroids); their composition varies according to the plant species, to the extraction method and the solvents used [2]. PCs from the HEs will be loaded in the SLNs and NLCs according to their lipophilicity (Figure 1).

## 4. Overview on SLNs and NLCs Entrapping Natural Bioactives for Skin Health

### 4.1. Delivery of Natural Bioactives from SLNs and NLCs

**Figure 2:**
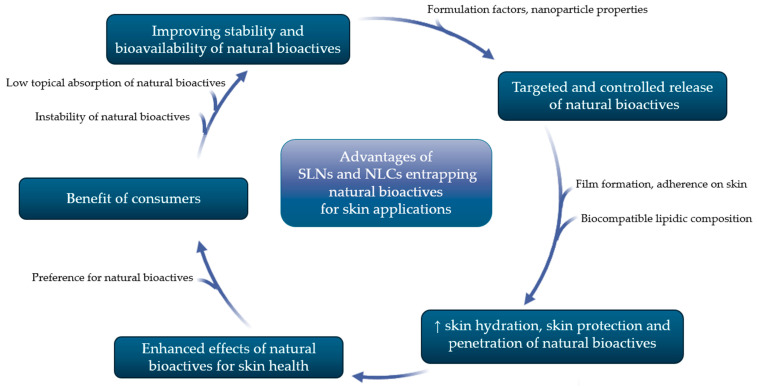
Summary of advantages of SLNs and NLCs entrapping natural bioactives for skin applications (image created using Biorender.com, accessed on 22 July 2024). ↑—increasing.

The advantages of entrapping natural bioactives in SLNs and NLCs are summarized in Figure 2 [23,36,37,38].

Natural bioactives are known to have poor bioavailability caused by their large molecular weight and poor skin permeability, along with their low stability to environmental factors. These drawbacks make natural bioactives good candidates for entrapment in nanoparticles, because they can improve their stability and bioavailability. Moreover, nanoparticles such as SLNs and NLCs may modulate the release of entrapped natural bioactives by adjusting the formulation factors and nanoparticles’ properties, to assure a targeted and controlled release. The increase in skin bioavailability is due to small particle size and also to their property of film formation, occlusion factor, and adherence on the skin. Thus, by increasing skin hydration and protection, the penetration of the nanoparticles loading natural bioactives is improved. Consequently, the enhanced effects of natural bioactives for skin health come as a huge benefit of consumers, as the preference for more natural products was recently observed. All in all, the innovative combination of natural bioactives extracted from plants with nanotechnology has opened new opportunities for developing cutting-edge topical products with enhanced skin delivery and, therefore, better anti-inflammatory, antioxidant, antimicrobial, or wound healing properties [23,36,37,38].

SLNs and NLCs seem to improve natural bioactives’ skin permeation by sticking to the skin surface, forming an adhesive film that increases skin hydration and promotes penetration of natural bioactives and of the carrier into the *stratum corneum* due to a reduced corneocyte packing and enlarged intercorneocyte space [39]. Here, lipid particles form a depot from which the natural bioactive is slowly released [40]. Surfactants’ presence in SLNs and NLCs may also increase the permeation of the loaded natural bioactives interfering with the lipid layer of *stratum corneum* through the fluidization and reorganization of its lipids [12]. The occlusion potential is the result of the surface lipidic film provided by the lipid nanoparticles and is related to the type of solid lipid and to the concentration of the surfactant used to formulate these nanocarriers [39].

The physicochemical properties of SLNs and NLCs, such as their shape, size, surface properties, and the diffusion coefficient, are important parameters which influence their permeation into the skin. The spherical shape, the small particle size, and the high surface area ensure their higher efficacy as delivery systems. The electrical charge of the lipid nanoparticles’ surface influences their aggregation and skin penetration pathways and diffusion [12,41].

### 4.2. The Advantages of Natural Bioactives Incorporation in SLNs and NLCs

The ability to entrap both hydrophilic and lipophilic actives, their remarkable biodegradability, biocompatibility, a good protection of the entrapped active, and good stability of nanoparticles, along with the controlled release of the entrapped active, represent some of the advantages of using SLNs and NLCs as carriers. The active ingredients loading capacity and their retention over time, lower in the case of SLNs, was improved for NLCs [42]. The improvement of the photostability of light-sensitive natural bioactives is another advantage of the encapsulation in SLNs and NLCs. Cordenonsi et al. demonstrated the increase in the photostability of fucoxanthin and conferred protection against its degradation after UVA light exposure, as compared to free fucoxanthin [43]. Mitri et al. showed the photoprotection of lutein incorporated in NLCs and SLNs, being higher for SLNs, at a radiation dose of 10 minimal erythema dose (MED) provided by a solar simulator [44].

These advantages led to the improvement of the biological effects. Thus, the enhancement of the antioxidant activity of the SLNs- and NLCs-loaded natural bioactives was demonstrated in a study conducted by Soldati et al., where higher antioxidant activity of SLNs-loaded resveratrol compared to free resveratrol and empty SLNs was observed through the DPPH (2,2-Diphenyl-1-picrylhydrazyl) test [45]. In another study, Okonogi et al. also observed a high antioxidant activity of NLCs containing lycopene compared to NLC base, after performing ABTS [2,2′-azino-bis (3-ethylbenzothiazoline-6-sulfonic acid)] and DPPH assays [46]. The antioxidant activity of sesamol was preserved and prolonged for up to 40 h after including sesamol in SLNs and NLCs, being higher in NLCs [31].

Gad et al. studied the wound healing effects of SLNs with *Chamomilla recutita* oil, observing the increase in wound area contraction, re-epithelization rate, collagen deposition, restoration of the normal skin architecture, and skin tensile strength [47]. Wound healing applications were also studied by Ghodrati et al., after incorporation of *Mentha piperita* EO in NLCs, by increasing neovascularization, fibroblasts infiltration, collagen synthesis, re-epithelization, and wound contraction ratio with better results after loading of EO in NLCs than free EO [48]. Navabhatra et al. also demonstrated the potential in wound care of these nanosystems, by loading *Cratoxylum formosus* extract in NLCs. It was found that the irritation potential of the free HE was decreased by loading the HE in NLCs [49].

Anti-inflammatory effects were also demonstrated by Gad et al., by increasing the transforming growth factor-β1 (TGF-β) levels and decreasing the interleulin-1β (IL-1β) levels and metalloproteinases-9/tissue inhibitor metalloproteinase-1 ratio. For all these assessments, better results were found for the creams with SLNs loading *Chamomilla recutita* oil followed by SLNs loading *Chamomilla recutita* oil and then the free oil [47]. Anti-inflammatory effects were also demonstrated by Rahmanian-Devin et al., when studying formulations to treat psoriasis-like lesions through histopathological assays on mice. SLNs loading noscapine considerably reduced tumor necrosis factor (TNF-α), transforming growth factor-beta (TGF-β1), and interleukin 17 (IL-17) levels compared to conventional cream with noscapine. Decreases in inflammation score, psoriasis area, and severity index (PASI), parakeratosis, hyperkeratosis, acanthosis were also observed [50].

The antimicrobial effects of natural bioactives have also been studied after incorporation in SLNs or NLCs. Ghodrati et al. showed the decrease in the total bacterial count in an in vivo infected wound model by application of *Mentha piperita* EO loaded in NLCs. It seems that the EO-loaded NLCs had the same antibacterial activity as the free EO [48]. After loading *Eucalyptus globulus* and *Rosmarinus officinalis* EOs in SLNs, Saporito et al. also noted the same inhibition of bacterial strains as the free EOs. Moreover, regarding the incorporation of EOs in NLCs, an increased inhibition compared to free EOs was observed [51]. Folle et al. found that NLCs loading thymol had higher antimicrobial activity on *Cutibacterium acnes*, but lower antimicrobial activity against *Staphylococcus epidermidis*, compared to free thymol [27].

Other notable applications of natural bioactives loaded in SLNs or NLCs and then incorporated in creams or gels consist of antiacne effects, where a decrease in the number of inflammatory acne lesions and in sebum rate were observed [52], along with an increase in skin hydration and skin elasticity [52,53], wrinkles reduction, and skin whitening [54].

Moreover, SLNs and NLCs are suitable carriers to deliver natural bioactives to skin appendages like hair follicles. Nanoparticles penetrate preferentially through the follicular pathway, facilitated by their smaller size [41]. The hair follicles serve as long-term reservoirs for topically applied substances, being able to store them for up to 10 days, which is 10 times longer than the reservoir of the *stratum corneum* [26,41]. Daneshmand et al. showed that SLNs with *Platycladus orientalis* L. extract allowed better absorption of cedrol, the marker molecule of this extract, to the hair follicle to prevent hair loss. A slow-release action was reported for SLN, as lipid nanodots were detected in the hair follicle even after 24 h of use [41]. Riangjanapatee et al. prepared *Camellia oleifera* seed oil-loaded NLCs, which showed better hair growth effect and a better localization of the active into the hair follicle than the oil itself [55].

Recently developed SLNs and NLCs entrapping HEs, Eos, or PCs for skin or hair applications are presented in Table 2. Their classification was made according to the type of nanosystems and the loaded natural bioactives, then specifying their composition, method of preparation, results of characterization, evaluation, effects reported, and applications. This classification may be used as a reference for future research in this innovative field.

**Table 2:** SLNs and NLCs entrapping EOs, HEs, and PCs for topical applications.

SLNs								
Entrapped Natural Bioactives	Lipids	Surfactants	Method of Preparation	Characterization	Applications	Evaluation: Samples and Test Type (In Vitro/Ex Vivo/In Vivo Studies)	Effects Reported	Ref.
**Oils and EOs**								
*Chamomila recutita* oil	Stearic acid	Tween^®^ 80	Hot homogenization.	Size 542.1–956.5 nm PDI: 0.273–0.723, ZP: (−25.6)–(−35.9) mV, Occlusion factor: 38.46–56.41%, Viscosity: 1.70–5.80 Pa s.	Wound healing	SNLs and cream with SLNs: In vivo wound healing study (Wistar rats), Ex vivo IL-1β and TGF-β1, MMP-9 and TIMP-1 levels and collagen deposition.	↑ Wound area contraction, ↑ re-epithelization grade, ↑ collagen deposition, skin architecture, ↑ tensile strength, ↑ TGF-β levels, ↓ IL-1β levels and MMP-9 activity (better results in cream with SLNs with oil > SLNs with oil > oil).	[47]
**HEs**								
*Platycladus orientalis* methanolic extract	Precirol^®^ Compritol^®^ Glucire^®^ Glyceryl monostearate	Tween^®^ 80, Labrasol^®^, Poloxamer^®^	High-shear homogenization and sonication.	Size: 34.32–1420 nm. PDI: 0.159–0.921. ZP: (−651)–(−4.35) mV. EE: 71%. Stability for 6 M.	Hair growth	SLNs: biofilm formation assay (*P. aeruginosa*, *P. mirabilis*, *E. coli*, *S.s aureus*, *E. fecalis*, *S. pneumoniae*), 5-α reductase activity.	↓ Biofilm development compared to control sample. ↑ 5-α reductase inhibitor activity.	[41]
*Withania somnifera* leaves ethanolic, hydroethanolic and aqueous extract	Compritol^®^ 888ATO, L-α-phosphatidylcholine	Tween^®^ 80, Span^®^80	Solvent injection method and sonication.	Size: 186.8–260.8 nm. PDI: 0.48–0.74. ZP: 16.7–27.3 mV. EE withaferin A: 35.4–70.0%. EE withanolide A: 81.5–95.3%. pH: 5.0–5.3. Stability for 3 M, room temperature.	Antimelanoma	SNLs: ex vivo skin diffusion and tape-stripping studies (human skin).	Relatively low concentrations of withaferin A and withanolide A diffused into the skin during 12 h. High EE, not correlated with a high release and skin permeation.	[56]
*Elaeis guineensis* fruits concentrated ethanolic extract	Glyceryl monostearate	Span^®^ 80, Tween^®^ 80	Hot homogenization.	Size: 609.70 nm. PDI: 0.22. ZP: −28.3 mV. pH: 4.77. Stability for 1 M (better at 4 °C than at room temperature).	Skin hydration	Creams with SLNs: skin hydration, TEWL, skin elasticity, melanin index, skin texture and satisfactory survey on human volunteers.	↑ Skin hydration, TEWL, cutaneous elasticity, and melanin index, skin moisturizing, ↓ wrinkles, ↑ elasticity, and skin whitening.	[54]
*Aloe vera* powder	Stearic acid, cetosteryl alcohol, and glyceryl monostearate	Tween^®^ 80	Modified solvent emulsification technique and sonication.	Size: 96.36–209.86 nm. PDI: 0.28–0.66. ZP: (−19.18)–(−11.02) mV. EE: 64.78 to 86.27%.	Photoprotective potential	Cream with SLNs: in vitro release profile dialysis bag (cellophane membrane), ex vivo permeation study (Albino rats’ skin) Determination of SPF: in vitro method and in vivo (Albino rats) Skin irritation test (Albino rats).	Improved topical retention of *Aloe vera* up to 12 h SPF 14.6–16.9 (in vitro) and 14.81 (in vivo) 80.10% permeation over 8 h. No irritation or sensitivity.	[57]
**PCs**								
Rutin	Beeswax, Carnauba wax	Tween^®^ 80, phosphatidylcholine	Hot melt microemulsion technique.	Size: 74.22 nm. PDI: 0.16. ZP: –53.00 mV. EE: 98.9%.	Photoprotective	SNLs: photochemopreventive effect against UVB radiation in ex vivo skin explants and 3D tissue engineering skin.	Efficient protection against UVB induced damage, inhibition of lipid peroxidation and metalloproteinase formation.	[58]
Noscapine	Precirol^®^, glyceryl monostearate, stearic acid, Campritol^®^ 888 ATO, cetyl palmitate	Poloxamer^®^ 188, Tween^®^ 80	High-shear homogenization method.	Size: 99.8–384 nm. PDI: 0.215–0.412. ZP: (−12.6)–(−32) mV. EE: 86.45–94.15%. Stability for 6 M, 30 °C, and 40 °C.	Psoriasis	SNLs: in vitro release profile, in vivo: inflammation degree, PASI on mice, histological analysis of ear samples, ELISA.	Cumulative release at pH 7.4 and 5.8 (72 h) ↓ inflammation degree and PASI, ↓ IL-17, TNF- α, TGF-β levels, ↑ IL-10 levels ↓ parakeratosis, hyperkeratosis, acanthosis.	[50]
Auraptene	Glyceryl monostearate Precirol^®^ ATO 5, stearic acid, glyceryl behenate	Poloxamer^®^ 188, Span^®^ 80	Hot homogenization and ultrasound method.	Size: 130.1–401.2 nm. PDI: 0.21–0.40. ZP: (−29.1)–19.4 mV. EE: 84.11%. Stability for 3 M, 25 °C, and 4 °C.	Anti-inflammatory	SNLs: in vitro release profile and skin retention study (mice skin). Anti-inflammatory activity (ear edema inflammation method), histopathological studies (mice) Skin sensitization study (Guinea pigs).	Biphasic release (burst release: 30–60 min, then sustained release until 24 h). ↑ cutaneous uptake and skin targeting. Improvement of the anti-inflammatory activity, no skin sensitization.	[59]
Tetrahydrocurcumin	Compritol^®^ 888 ATO	Tween^®^ 80, phosphatydylcholine	Microemulsification technique.	Size: 96.6 nm. PDI: 0.252. ZP: −22 mV. EE: 69.56% Stability for 2 M, 4 °C, and 40 °C.	Anti-inflammatory, wound healing	SLNs and hydrogel with SNLs: in vitro release profile (dialysis membrane). Ex vivo permeation studies (pig ear skin). Hydrogel with SNLs: acute dermal irritation studies.	↑ Drug release for 24 h. ↑ Skin permeation. No erythema or edema.	[60]
Curcuminoids	Beeswax	Tween^®^ 80, phosphatidylcholine	Hot melt emulsion technique, high-shear homogenization.	Size: 32.7–481.9 nm. PDI: 0.179–1.000. ZP: (−34.0)–(−21.2) mV. EE curcumin: 41.4–62.0%. EE total curcuminoids: 31.0–58.78%.	Anti-inflammatory, radiodermitis	SLNs: in vitro release profile. Gel with SNLs: ex vivo permeation study (pig ear skin), retention in the epidermis and dermis.	Sustained and slow release for 16 h, reduced permeation in epidermis/dermis for 18 h.	[61]
Curcumin	Precirol^®^ ATO 5	Tween^®^ 80, Span^®^ 80	Pre-emulsion technique and sonication.	Size: 51.8–107.0 nm. PDI: 0.211–0.462. EE: 85–93%. DL: 5.05–9.51%.	Contact dermatitis (pigmentation, irritation)	Gel with SNLs: ex vivo drug diffusion, drug deposition studies, antioxidant activity (DPPH assay), in vitro tyrosinase inhibition assay skin irritation test, efficacy against irritant contact dermatitis (BALB/c mice ears).	Controlled drug release up to 24 h, potential in skin depigmentation, ↑ antioxidant activity, proficient suppression of ear swelling and reduction in skin water content in the BALB/c mouse.	[62]
Resveratrol	*Theobroma grandiflorum* seed butter	Pluronic^®^ F-127	High shear homogenization technique and sonication.	Size: 195.30 nm. PDI: 0.16. ZP: −19.54 mV. DL: 3.36%. EE: 74.12%.	Antioxidant	SLNs: in vitro release profile (polysulfone membrane), in vitro permeation (human skin), antioxidant activity (DPPH assay).	Slow and sustained release for 24 h. ↑ Concentration of resveratrol retained in the skin.	[45]
**NLCs**								
**Oils and EOs**								
*Rosmarinus officcinalis* EO	Cetyl palmitate	Oleth-20, Glyceryl Oleate	Phase inversion temperature method.	Size: 26.90–171.70 nm. PDI: 0.171–0.495. ZP: (−1.55)–(−2.19) mV. Stability for 2 M, room Temperature.	Skin hydration	Gels with NLCs: skin hydration and elasticity on human volunteers.	↑ Skin hydration and elasticity.	[53]
*Menta piperita* EO	Precirol ^®^ -ATO 5, Miglyol ^®^ -812	Poloxamer^®^	Hot melt homogenization technique.	Size: 40–250 nm. PDI: ~0.4. ZP: (−10)–(−15) mV. EE 93.2%. DL 9.3%.	Antibacterial, wound healing	NLCs: wound area rate, histopathological studies, molecular analysis, antimicrobial activity, total tissue bacterial count.	↑ Wound contraction rate, fibroblast infiltration, collagen deposition, and re-epithelialization, positive effects on the FGF-2 and EGF mRNA levels expressions, antibacterial activity against *S. epidermidis*, *S. aureus*, *L. monocytogenes*, *E. coli*, and *P. aeruginosa.*	[48]
*Piper aduncum* oil	Caprylic/capric triglycerides, *Theobroma grandiflorum* (Cupuaçu) butter	Tween 20^®^, Span 80^®^	Ultra-turrax homogenization and high-pressure homogenization.	Size: ~130 nm. PDI: 0.17. ZP: −40.50 mV. EE: 89.42%.	Anti-inflammatory	NLCs and hydrogels with NLCs: In vitro release profile (cellulose ester membrane), in vitro permeation/retention studies on porcine skin, HET-CAM Toxicity Test.	Controlled and constant release: initial burst for 2 h, then slower and continuous release for 12 h Delivery of dillapiole to dermis, low irritation potential.	[63]
*Camellia oleifera* seed oil	Olivem^®^ 1000	Tween^®^ 80, Varisoft^®^ 442	High-speed homogenization technique.	Size 80–290 nm. PDI 0.15–0.8. EE 96.26%. Stability for 3 M, 4 °C, 25 °C, and 40 °C.	Hair growth	NLCs: In vitro cell viability (HFDP cells).	Certain NLCs ↓ and other ↑ cell viability, depending on the surfactant and on the physicochemical properties. Hair growth stimulating effect and a better localization of the active into hair follicle.	[55]
*Rosa canina* oil, *Nigella sativa* oil, *Daucus carota* extract, *Calendula officinalis* extract	Glycerol monostearate, cetyl palmitate	Tween^®^ 20, Poloxamer^®^ 188, phosphatidylcholine	Melt-emulsification and high-pressure homogenization.	Size: 118–158 nm. PDI: 0.108–0.189. ZP: (−41.6)–(−64.3) mV. EE β-carotene: 94.37–97.88%. EE azelaic acid: 91.76–93.48%.	Antioxidant, anti-inflammatory, antiacne, skin hydration	Hydrogels with NLCs: in vitro release profile (cellulose nitrate membrane), in vitro antioxidant action (chemiluminescence and TEAC methods), antimicrobial activity. Studies on cell lines: cytotoxicity, in vitro anti-inflammatory activity, in vivo hydration degree, antiacne potential (human volunteers), anti-inflammatory (Wistar rats).	Sustained release during for 8 h, ↑ antioxidant activity, antimicrobial activity on *S. epidermidis*, *C. acnes*, *C. albicans*. Biocompatibility with fibroblasts, ↓ IL-1β and TNF-α, ↑ skin hydration and skin elasticity, ↓ number of inflammatory acne lesions, ↓ sebum rate.	[52]
**HEs**								
*Cratoxylum formosum* leaf ethanolic extract, lyophilized	Glyceryl behenate, glyceryl monostearate, caprylic/capric triglycerides	Tween^®^ 80, Poloxamer^®^ 188	High-shear ultrasonic Homogenization.	Size: 57.68–489.33 nm. PDI: 0.16–0.45. ZP: (−15.06)–(−5.76) mV. EE: 40.30–80.76%. Stability for 3 M, 4 °C, 25 °C, and 40 °C.	Skincare	NLCs: in vitro release profile (dialysis bag), skin permeation (porcine ear skin), cell viability (HDFn).	↑ Skin absorption and biocompatibility, ↓ irritation potential than the free extract solution.	[49]
**PCs**								
Curcumin	Medium chain triglycerides, glyceryl monostearate, Span^®^ 80	Tween^®^ 80	High-pressure homogenization.	Size: ~200 nm. PDI: ~0.25. ZP: ~>−20 mV. EE: >90%. Stability for 4 M, 4°, 25°, and 37 °C.	Skin burns	NLCs: in vitro release profile (dialysis bags), ex vivo skin permeation and retention studies (porcine ear skin), cell viability on human skin fibroblasts (HFF-1 line), antimicrobial activity.	Release of curcumin in 72 h (51.64%). No toxicity on cells. ↑ Antimicrobial activity on *Pseudomonas aeruginosa.*	[64]
Curcumin and caffeine	Stearic acid, oleic acid	Soya lecithin, polyvinyl alcohol	Hot homogenization and ultrasonication.	Size: 98–169 nm. PDI: 0.23–0.58. EE: 44.14–63.92%.	Psoriasis	NLCs: in vitro release profile (dialysis bag), gels with NLCs: in vitro drug diffusion studies (cellophane dialysis membrane), in vivo studies (mice with induced psoriasis), skin irritation, skin inflammation, ex vivo permeation studies, PCs retention in the skin layers.	Initial burst release followed by prolonged release of drug for 12 h, gels with NLCs: compatibility and nonirritant effect. ↓ Inflammation, ↑ efficacy of psoriasis treatment.	[65]
Thymol	Glyceryl behenate, PEG-8, caprylic/capric triglycerides,	Tween^®^ 20	High-pressure homogenization and sonication.	Size: 123.8–360.0 nm. PDI: 0.113–0.359. ZP: (−13.02)–30.81 mV. EE: 76.38–81.41%. Stability for 6 M, room temperature.	Anti acne	NLCs and gels with NLCs: in vitro release profile (methylcellulose membranes), cell viability on HaCaT, ex vivo skin permeation studies (human skin explants), antimicrobial activity.	Prolonged release for 72 h cytotoxic at high doses, at lower doses and removal of excess free Tween^®^ 20 from the formulation, cell viability ↑ significantly ↑ antimicrobial activity on *C. acnes*, *S.s epidermidis.*	[27]
Naringenin, nordihydroguaiaretic acid, kaempferol	Gelucire^®^ 50/13, Apifil^®^, Miglyol^®^ 812, Labrafac^®^ WL1349	Tween^®^ 80, Pluronic^®^ F-127	High shear homogenization and sonication.	Size: 176–213 nm. PDI: 0.205–0.234. ZP: (−22.9)–(−8.9) mV. EE: 89.9–98.8%. Stability for 1 M.	Antioxidant, cancer prevention	NLCs: in vitro release profile, antioxidant activity (DPPH assay), cell viability and antioxidant activity (HaCaT).	Slow and sustained release for 19 days. No significant cytotoxicity. Good antioxidant activity.	[66]
Lycopene	*Citrus sinensis* wax, *Oryza sativa* bran oil	Eumulgin^®^ SG	High pressure homogenization.	Size: 158–166 nm. PDI: 0.13–0.15. ZP: (−74.6)–(−74.2). pH: 6.6, EE: >99%. Stability for 4 M, 4 °C, 30 °C, and 40 °C.	Antioxidant	NLCs: in vitro release profile a.	Biphasic release profile: fast release for 6 h, then sustained release for 18 h.	[46]
**SLNs and NLCs**								
**Oils and EOs**								
*Eucalyptus globulus* or *Rosmarinus officinalis* oils	*Theobroma cacao* butter, *Olea europaea* oil, *Sesamum indicum* oil	L-α-phosphatidylcholine	High shear homogenization and sonication.	Size: 50–60 nm. PDI: ~0.5. ZP: −22.07 mV. Good bioadhesive properties. Stability 3 M, 2–8 °C.	Wound healing	SLNs and NLCs: antimicrobial activity cytocompatibility, in vitro proliferation enhancement, and wound healing properties in human dermal fibroblasts, in vivo wound healing efficacy (Wistar rats).	Same inhibition on *Staphylococcus aureus* and *Streptococcus pyogenes* as the free oils for SLNs and ↑ inhibition than free oils for NLCs, good biocompatibility, ↑ cell proliferation.	[51]
**PCs**								
Orobol	*Theobroma cacao* butter, *Butyrospermum parkii* butter, Capmul^®^ MCM EP	Transcutol^®^ HP, Tween^®^ 20	Hot homogenization and sonification.	Size: 133–498 nm. PDI: 0.140–0.211. EE: 95.7–97.2%. DL: 0.91–0.97%. Stability 28 days, room temperature.	Antiaging	SLNs and NLCs: deposition study (Strat-M membranes and human cadaver skin), skin irritation (human volunteers).	Better results on NLCs: ↑ membrane/skin deposition of orobol, no significant skin irritation.	[67]
Sesamol	Compritol^®^888 ATO, Miglyol^®^ 812, *Sesamum indicum* oil	Poloxamer^®^ 188	Hot homogenization and sonification.	Size: 169.2–224.7 nm. PDI: 0.277–0.317. ZP: (−35)–(−38) mV. EE: 78.5–91.2%.	Antioxidant	Hydrogel with SLNs and NLCs: in vitro percutaneous absorption (human skin), in vitro skin permeation (human SCE membranes), in vitro antioxidant efficiency (ORAC assay).	↓ Permeation from NLCs, controlled diffusion through the skin, prolonged antioxidant activity.	[31]
Resveratrol	Octyl and decyl glycerate, glycerol monosterate	Cremophor^®^ A25 and A6	Hot high-pressure homogenization.	Size: 297.9–336.4 nm. PDI: 0.352–0.405. ZP: 4.9–6.8 mV. EE: 94.8–96.2%. Stability for 6 weeks, 2–6 °C.	N.A.	SLNs and NLCs: in vitro release profile (dialysis bags), in vitro penetration study.	Biphasic drug release profile (burst release at the initial stage, then controlled release for 24 h) ↑ Penetration of SLNs compared. to NLCs.	[32]

↑—increasing, ↓—decreasing, HEs—herbal extracts, EOs—essential oils, PCs—phytocompounds, N.A.: not available, ZP—zeta potential, EE—encapsulation efficiency, DL—drug loading, M—months, HDFn—primary human dermal fibroblasts, PIT-phase inversion temperature, DPPH (2,2-Diphenyl-1-picrylhydrazyl), TEWL—transepidermal water loss, TEAC—Trolox Equivalent Antioxidant Capacity, HSV1—Herpes simplex virus 1, SPF—sun protection factor, LMW—low molecular weight, HFF—human foreskin fibroblasts, Hen’s Egg Chorioallantoic Membrane Test (HET-CAM, Eumulgin SG (sodium stearoyl 69 glutamate), SPF—sun protection factor, PASI—Psoriasis Area and Severity Index, ELISA—enzyme-linked immunosorbent assay, Substances: Precirol^®^-ATO 5—Glyceryldistearate, Miglyol^®^-812—caprylic/capric triglycerides, Labrasol^®^-PEG-8 caprylic/capric glycerides, Compritol^®^ CG 888 ATO—Glyceryl behenate, Transcutol P^®^—Diethylene glycol monoethyl ether, Poloxamer^®^ 188—a -Hydro-o-hydroxypoly(oxyethylene)poly(oxypropylene) poly-(oxyethylene), Tween^®^ 80—polyoxyethylene sorbitan monooleate, Span^®^ 80-Sorbitan monooleate, Olivem^®^ 1000—Cetearyl olivate, sorbitan olivate, Varisoft 442 100P.-Quaternium-18 Miglyol^®^ 812-Caprylic/Capric Triglyceride, Capmul^®^ MCM EP-Glyceryl Caprylate/Caprate, Cremophor^®^ A25—Ceteareth 25, Cremophor^®^ A6—ceteareth 6, stearyl alcohol.

To address skincare applications related both to dermatology and cosmetics such as wound healing, antibacterial, anti-inflammatory, antimelanoma, photoprotective, skin hydration, skin whitening, and the treatment of psoriasis, acne, and hair loss, a variety of EOs, oils, HEs, and PCs have been loaded in SLNs and NLCs. Of the many encapsulated natural bioactives, curcuminoids were often used, due to their multifaceted potential therapeutic properties, as well as their poor bioavailability, which makes them interesting to include in SLNs and NLCs [68]. A variety of lipids were used to obtain SLNs and NLCs, of which the most common was glyceryl monostearate, stearic acid, and Compritol^®^ 888 ATO (Glyceryl behenate). Regarding the NLCs, the most used liquid lipids were caprylic/capric triglycerides and various natural oils. The lipids were chosen as a function of their compatibility with the bioactive ingredient and biocompatibility with the skin [34]. The most used surfactants were Tween^®^ 80 (polyoxyethylene sorbitan monooleate), Poloxamer 188 (a-Hydro-o-hydroxypoly(oxyethylene)poly(oxypropylene) poly-(oxyethylene), and Span^®^ 80 (Sorbitan monooleate). Due to their amphiphilic structure, surfactants lower the surface tensions and promote partitioning into hydrophilic and hydrophobic groups from the dispersion, preserving the stability of the SLNs and NLCs. The selection of surfactants must be chosen to avoid irritation and sensitivity of the skin [34]. Methods of preparation of SLNs and NLCs such as hot homogenization and high-shear homogenization, usually followed by sonication, are commonly employed.

The characterization of SLNs and NLCs loaded with natural bioactives is important to ensure the efficacy and the delivery to the desired skin layer. Further, the parameters used for the characterization of nanosystems presented in Table 2 are discussed below. As can be seen in Table 2, the particle sizes of SLNs and NLCs entrapping natural bioactives ranged from 32.7 to 1420 nm. SLNs and NLCs typically have diameters between 40 to 800 nm, which enables them to attach to the *stratum corneum*’s lipid matrix and increase the quantity of active ingredients that reach the skin’s deeper layers. In general, nanosystems with a diameter of 600 nm or higher are unable to deliver loaded bioactive substances to deeper skin layers; they tend to remain on or in the *stratum corneum*, where they can form a lipid layer after drying [26]. It is generally acknowledged that the particle size should be smaller than 300 nm to allow efficient skin penetration, while maximum deposition of actives in both viable dermal and epidermal layers is obtained when particle size is below 70 nm [23]. It was reported that particles with size below 200 nm or 300 nm can also enter the skin via the hair follicles [25,26]. The particle size of the SLNs and NLCs can be influenced by the lipid matrix’s composition. It was reported that size decreased with increasing oil content of the NLCs, probably due to lower viscosity of the melted droplets, thus easing dispersion and resulting in smaller sizes [44]. The particle size of SLNs and NLCs may also depend on the concentration of surfactant required to form an adsorption monolayer on the particles [57]. It was found that particle size significantly decreased with increasing concentration of Tween^®^ 80 [62]. Another parameter important for particle characterization is the homogeneity of particle size, evaluated through the determination of the polydispersity index (PDI). The PDI of SLNs and NLCs with natural bioactives varied widely between 0.16 and 1.00 (Table 2). Particle monodispersity is obtained when PDI has values lower than 0.3. PDI can be narrowed and the particle size of SLNs and NLCs can be reduced by sonication or by increasing the number of homogenization cycles when obtained by the high-pressure homogenization method. However, the application of too many cycles may determine the agglomeration of coalescence of particles, due to the additional energy input and higher kinetic energy [23,44,62]. The zeta potential (ZP) is influenced by the net charge of the lipids and surfactants from the structure of the SLNs and NLCs. If the particles are uncharged, so the closer the ZP values are to zero, they are prone to flocculation. Ideally, ZP should reach values below −25 mV or greater than 25 mV, but, as it seems from the research results, this is quite difficult to achieve. For SLNs and NLCs loading natural bioactives, ZP varied between −651.0 to 27.3 mV, with most of the values being below −25 mV (Table 2), as is necessary for ensuring a high stability, due to the repulsive forces that have the natural tendency to aggregate [23]. Encapsulation efficiency (EE) is represented by the amount of entrapped active ingredient from the total amount of the active which was initially added to the dispersion of natural bioactives [34]. For SLNs and NLCs entrapping natural bioactives, EE ranged between 31.0% and 99% (Table 2), indicating the effectiveness of different preparation methods in their encapsulation. Drug loading (DL) or loading content of natural bioactives is calculated by dividing the amount of entrapped natural bioactives by the amount of lipid used [45]. DL varied from 0.91 to 9.51% (Table 2), even though it was not reported in most studies. SLNs and NLCs with natural bioactives exhibited physical stability for a period between 1 and 6 months, analyzing the parameters presented above. They were stable when stored at room temperature and had even better stability at 4 °C (Table 2).

The kinetic studies on SLNs and NLCs containing natural bioactives were performed in vitro using synthetic membranes or ex vivo studies using skin explants (Table 2). These studies have documented the release profile, percutaneous absorption, deposition in different skin layers, or permeation characteristics of these parameters. The most performed test was the in vitro release profile assessment, which demonstrated the sustained or prolonged release of the bioactives [32]. The bioactives’ solubility in the lipid matrix is the first major factor influencing their release [43,46]. Additionally, the increased concentration of lipids and surfactants in the SLNs and NLCs formulation ensures a faster diffusion of loaded bioactives and, consequently, a better release [57]. Interestingly, some studies revealed a biphasic pattern, a relatively fast release during the first hours followed by a sustained release during the next many hours [46,59,63]. A fast release at the initial stage was due to the high amount of lipophilic bioactive dissolved in this liquid lipid shell. This phenomenon was found for NLCs, due to some lipids that can lead to accumulation of the lipophilic bioactive in the outer shell after lipid crystallization, because of the distribution of liquid lipid in the solid lipid matrix and their different melting behavior. The wide surface area of the NLCs and a high diffusion coefficient resulting from the low viscosity of the matrix are the other elements that promote the observed quick release in the first stage. Additionally, it has been found that decreasing the liquid lipid ratio can accelerate the release rate during the burst release stage [32]. In most studies, a sustained release for 72 h was reported, while in the study reported by Gonçalves et al., a slow and sustained release for 19 days was observed [66].

Skin permeation and skin retention studies were performed using dialysis method with Franz diffusion cells and ear porcine skin as the membrane, comparing nanoemulsions (NEs) and NLCs loaded with curcumin. The results showed that curcumin from NLCs was retained in the epidermis at levels 1.25 times higher (µg/cm^2^) than from NEs, while in the dermis its concentration was 1.37 times lower than from NEs. No curcumin was detected in the acceptor fluid, indicating that these nanocarriers were preferentially retained in the skin layers, without penetrating the systemic circulation [64]. The release of dillapiole from NEs and NLCs loaded with *Piper aduncum* EO rich in dillapiole was performed using Franz diffusion cells and a synthetic cellulose ester membrane. The percentage of the released dillapiole from NLCs was about three times lower than from NEs, probably due to the solid core of NLCs. The skin permeation and retention studies performed using porcine ear skin showed that dillapiole has a good ability to penetrate the deeper layers of skin, as it was not retained in the epidermis, but was retained in an important amount in the dermis, where an important cumulative amount permeated was determined both from NEs and NLCs. This finding showed an important potential of these nanocarriers for the management of topical inflammatory diseases [63]. The release profile and the skin retention ability were also studied using Franz diffusion cells and methylcellulose membranes, comparing an aqueous formulation, NLCs encapsulating thymol, and gels containing NLCs entrapping thymol. The aqueous solution of thymol reached a steady state around 12 h, while both NLCs and gel formulations of NLCs exhibited a prolonged release for 72 h. Ex vivo permeation studies realized using human abdominal skin obtained from plastic surgery indicated that thymol from the aqueous formulation reached the systemic circulation faster and its retention at skin levels was lower than from the NLCs carrier. This finding suggested that NLCs encapsulation of thymol was a more effective approach for the therapy of topical conditions [27].

Other studies, conducted in vitro on cell cultures (e.g., fibroblasts, keratinocytes) and in vivo studies in animals (mice or rats) or human volunteers, indicated enhanced biological effects when using SLNs and NLCs compared to free bioactives (Table 2).

With regard to the potential drawbacks of these nanosystems, there were studies that reported cytotoxicity of NLCs in HaCaT cells, depending on surfactant type and increasing with its concentration, knowing that some surfactants may have cytotoxic potential at high doses. For example, in a study conducted by Folle et al., the cell viability increased after removing the free Tween^®^ 20 (Polyoxyethylene (20) sorbitan monolaurate) from the samples. Other authors have stated that the cytotoxicity of SLNs and NLCs may be attributed also to lipid peroxidation and generation of oxygen free radical species, depending on the cell type and concentration used [27,69,70].

## 5. Formulation of SLNs and NLCs into Semisolid Systems and Use as Dispersions: Revealing Their Properties for Specific Applications

Products applied on skin must have desirable viscosity, texture, and sensory properties according to their specific applications and conditions of usage. For example, in some applications when large skin surfaces are targeted, low viscosity is desired, ensuring the fluidity needed for an easy application [24], while in other cases, when a small skin surface is targeted, a reduced viscosity can be less attractive or even a disadvantage [43].

SLNs and NLCs can be used as dispersions, per se, or incorporated into semisolid formulations, e.g., creams or gels.

### 5.1. SLNs and NLCs Used as Dispersion Per Se

SLNs and NLCs may have good bioadhesive properties, probably determined by their flexibility, which could promote interaction with the biologic substrate and the consequent bioadhesive joint formation. To be used in topical administration, this represents an important feature to allow an intimate contact between formulation and the skin, to assure treatment efficacy. The bioadhesive properties can be evaluated using a texture analyzer and a biologic substrate [51].

When nanoparticles come into contact with skin, they generally attach excellently due to a film-forming property generating an occlusive film (at a minimal concentration of 2–4% of NLCs) which promotes the penetration of actives. Additionally, the lipids from the SLNs and NLCs structure can interact with matrix lipids from the stratum corneum when the melting point of the lipids is close to the temperature of the skin (t < 32 °C). If the melting temperature of the lipids is higher, the particles remain on the skin surface, and they can be slightly deformed during application [39]. The film-forming property results from the adsorption of these particles on skin, which lowers the surface free energy, and, hence, the thermodynamic driving force of the resulting system. Another explanation of this phenomenon is represented by the property of ultrafine materials to form high van der Waals forces between the particles and contact surface. If a monolayer of lipophilic coating occludes the skin, the evaporation-induced loss of moisture may be postponed. Moreover, the pressure obtained during skin application causes the fusion of the particles, forming a dense coating on the skin, with this fusion also being supported by the capillary forces involved in water evaporation [24,46]. The occlusive effect depends on the lipids’ ratio, the sample volume of the product applied, particle size, and crystallinity of particles [26]. Depending on the particle size of the SLNs and NLCs, the moisture barrier’s properties reveal different degrees of occlusion. Thus, the barrier against evaporation increases with a decrease in their particles size, while the evaporated amount of water will increase with their particle size [24]. An increase in the occlusion factor over time has been demonstrated as well [46]. The ability to form an occlusive film by the SLNs and NLCs or their formulations (after including in gels and creams) can be measured using an occlusion study [24,46,47,57,60,62]. Moreover, it was shown that SLNs and NLCs provide good skin lubrication and smoothness due to their spherical-like shape. The lubricating effect assessment could be conducted using rheological analysis [24].

Using different types and concentrations of lipids and surfactants, the formulators can adjust the SLNs and NLCs properties depending on their usage particularities [1,24]. The choice of the ingredients should start with compatibility studies between the active substances and excipients [32,66]. DSC (differential scanning calorimetry analysis) and FTIR (Fourier transform infrared spectroscopy) of the lipids, surfactants, and bioactives intended to be used, as well as their physical mixture, can be evaluated to observe the interactions between the ingredients [65]. The study of the influence of multiple formulation variables (type and concentration of lipids, surfactants, and active substances contained) on the SLNs and NLCs properties (e.g., particle size, PDI, zeta potential, encapsulation efficiency, etc.) can be assessed using a design of experiment approach, a systematic method with the advantages of minimal number of experimental runs and reduced consumption of raw materials [27,28,49,50,59,62,65,71,72]. Moreover, process formulation factors can be also varied (e.g., homogenization time) and then their influences on the quality characteristics of the SLNs and NLCs can be studied [61].

### 5.2. SLNs and NLCs Used after Incorporation in Topical Vehicles

Table 3 presents an overview of the SLNs and NLCs entrapping HEs, oils, Eos, and PCs included in topical application systems. To facilitate topical application, SLNs and NLCs can be dispersed into several vehicles to form semisolid formulations, also improving the acceptability and user compliance, and even increasing their stability [27,43,60]. Thus, a convenient choice for vehicles can be represented by gels or hydrogels, e.g., Carbopol^®^ (Carbomer) or cellulose derivates gels, because of the forming gel threads with SLNs and NLCs embedded within them. In a study conducted by Folle et al., NLCs were included in three different types of gels; carbomer and hydroxypropyl methyl cellulose (HPMC) gels containing NLCs provided an easy spreading application onto the skin and Pluronic^®^F127 (Poloxamer 407, Oxirane, methyl-, polymer with oxirane) gel containing NLCs provided a more dried touch and non-easy spreadable formulation. The investigation of transmission electron microscopy (TEM) images revealed that gel threads had a lipidic appearance, where the NLCs were embedded. It presented an amorphous lipidic (waxy-like) appearance, like an emulgel (nongreasy, easy spreadable, and easily removable). The NLCs dispersion was found to be stabilized after inclusion in gels, making them suitable for storage to avoid creaming or flocculation phenomena. Carbopol^®^ and HPMC gels loaded with NLCs had higher and similar amount of active compound retained inside the skin compared to Pluronic^®^F127 gels containing NLCs, which exhibited a very slow penetration rate of the natural bioactive. Carbopol^®^ gels containing NLCs have been shown to be highly retained in skin tissues, provided a low permeation rate and a prolonged release up to 72 h [27]. Moreover, SLNs and NLCs can be formulated as hydrogels by including hydroxyethyl cellulose or Carbopol ^®^ in the dispersion. In the study conducted by Caneiro et al., the resulting hydrogels-thickened NE and NLCs exhibited pseudoplastic behavior and bioadhesive properties. The hydrogels-thickened nanosystems proved to be more bioadhesive since the work of adhesion and the force of detachment were about eight times and three times higher in comparison with the NE and NLCs without hydroxyethyl cellulose. Additionally, a lower retention of active substance in the dermis was observed from hydrogels-thickened nanosystems in comparison with NE and NLCs without hydroxyethyl cellulose. Due to the higher viscosity of the system, the hydrogels-thickened nanosystems may hinder the delivery of active compounds, because they should leave the polymeric network and then penetrate the skin [63]. In another study by Kakkar et al., Carbopol^®^ gel was added in the SLNs dispersion to obtain a hydrogel. The release of the active from the Carbopol ^®^ gel-loading SLNs and SLNs dispersion was almost 16–17 times higher than the free substance hydrogel during the ex vivo permeation studies [60]. Gels containing Rosmarinus officinalis EO-loaded NLCs showed a significant increase in skin hydration and a lesser increase in skin elasticity in comparison with gels containing free Rosmarinus essential oil [53]. In a study conducted by Riangjanapatee et al., NLCs loading Camellia oleifera seed oil were incorporated in serums, which reduced the sticky, oily, and greasy feeling, while ensuring an easier application (better spreadability and reduction in firmness) [55].

SLNs and NLCs can also be included in creams. Their incorporation requires careful formulation to ensure uniform distribution, prevent phase separation, and ensure their overall stability. SLNs and NLCs are usually incorporated in the final step, by gently stirring them into water-based products, when the temperature is around 30 °C [39]. The stability of topical products with SLNs and NLCs was evaluated by specific methods. For example, in the study conducted by Plyduang et al., SLNs loading *Elaeis guineensis* fruit extract included in creams were stable after six cycles of freeze–thaw and after storage at 30 °C/75% RH for 6 months, in terms of pH, viscosity, and PCs content (tocopherol, tocotrienol, and beta-carotene) [54].

**Figure 3:**
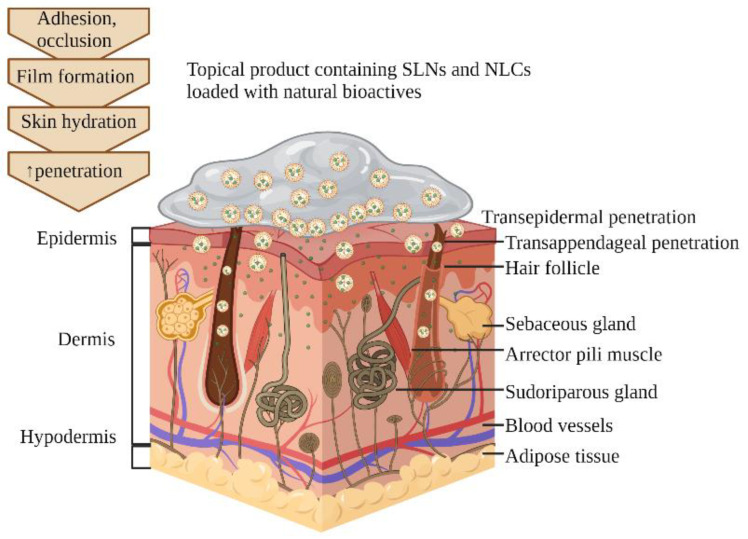
Skin bioavailability enhancement of the natural bioactive substances loaded in SLNs and NLCs from a topical product (image created using Biorender.com, accessed on 2 August 2024).

Figure 3 schematically represents the skin bioavailability enhancement of the natural bioactive substances loaded in SLNs and NLCs from a topical product, as described above, in Section 5.1 and Section 5.2. The adhesion and occlusion formed by SLNs and NLCs included in a topical product determine film formation, which increases skin hydration and, furthermore, increases the skin penetration of SLNs and NLCs and the natural bioactives contained. The skin absorption of SLNs and NLCs through transepidermal and transappendageal routes are also illustrated.

**Table 3:** Overview of SLNs and NLCs loading HEs, EOs, or PCs, included in topical application systems.

Entrapped EOs, HEs, PCs	Concentration of the Natural Bioactives in the Final Topical System	Composition of the Topical System	Ref.
**SLNs**			
*Chamomilla recutita* oil	N.A.	Camisan^®^ cream (EIPICO, Egypt)	[47]
*Rosmarinus officinalis* essential oil	N.A.	Carbopol^®^ hydrogel (Carbopol^®^ Ultrez 21^®^, TEA, Imidazolidinyl urea, Methylchloroisothiazolinone, Methylisothiazolinone)	[53]
*Aloe vera* powder	N.A.	Sunscreen cream (Cetosteryl alcohol, Stearic acid, TEA, Glycerol, Liquid paraffin)	[57]
*Elaeis guineensi*s fruit extract	3%	Day and night creams (Liquid paraffin, White soft paraffin, Isopropyl myristate, Glyceryl monostearate, Cetyl alcohol, Stearic acid, Carbomer 940, Glycerin, Propylene glycol, Polysorbate 80, Tocopherol acetate, Disodium EDTA, Phenoxyethanol, Titanium dioxide, Octyl methoxycinnamate, Grape seed extracts)	[54]
**NLCs**			
*Piper aduncum* oil	N.A	Hydroxyethyl cellulose hydrogels	[63]
Curcuminoids	N.A.	Natrosol^®^ gel (Via Pharma Ltd.a, Brazil)	[61]
Sesamol	0.1%	Sodium polyacrylate hydrogels	[31]
*Rosa canina* oil, *Nigella sativa* oil, *Daucus carota* extract, *Calendula officinalis* extract	6% *Daucus carota* extract/*Calendula officinalis* extract, 2% azelaic acid and 12% *Rosa canina* oil/*Nigella sativa* oil	Carbopol^®^ hydrogel (Carbopol^®^ 940, ethanol, glycerin, TEA)	[52]
Thymol	0.1%, 0.25%	Carbopol^®^ hydrogel (Carbopol^®^ 934, glycerin, propylene glycol) Hydroxypropyl methylcellulose gel (HPMC, glycerin, propylene glycol) Pluronic^®^ gel (poloxamer 407/Pluronic^®^F127, glycerin, propylene glycol)	[27]
Tetrahydrocumarin	0.2%	Carbopol^®^ hydrogel (Carbopol^®^ 934, TEA)	[60]
Curcumin	N.A.	Carbopol^®^ hydrogel (Carbopol 940^®^, propylene glycol, TEA, methyl and propylparaben)	[62]
Curcumin and caffeine	N.A.	Carbopol gel (Carbopol-934, propylene glycol, TEA, propyl paraben)	[65]
*Camellia oleifera* seed oil	N.A.	Serum (Ammonium acryloyldimethyltaurate/VP copolymer, glycerin, phenoxyethanol, and chlorphenesin)	[55]

HEs—herbal extracts, EOs—essential oils, PCs—phytocompounds, N.A.—not available, Carbopol^®^—Carbomer, TEA—triethanolamine, EDTA—Ethylenediaminetetraacetic acid.

## 6. Key Features of Commercial Products Incorporating SLNs and NLCs

The range of commercially available products with SLNs and NLCs has significantly widened in recent years due to their functional benefits. From the perspective of the cosmetic industry, the cost-effective manufacturing process by high-pressure homogenization and the ease of incorporating nanoparticles in the final formulations provide significant advantages. The possibility to use various natural oils such as *Argania spinosa* kernel oil, *Aleurites moluccana* seed oil, *Macadamia ternifolia* seed oil, *Olea europea* oil, *Ribes nigrum* seed oil, and *Prunus amygdalus* dulcis oil, as well as herbal extracts (*Panax ginseng* root, *Equisetum arvense*, *Camelia sinensis* leaf, *Viola tricolor* extract) or different active ingredients (coenzyme Q10, fatty acids), allows for personalized formulations and additional marketing claims [37,39]. SLNs and NLCs improve the efficacy and tolerance of sunscreens, acting as penetration enhancers, thus allowing for smaller ratios of actives and minimizing side effects. They provide excellent photoprotective action and enhanced active ingredient EE, depending on the choice of the lipids used [73].

The first cosmetic product incorporating NLCs (Cutanova Nanorepair Q10) was launched in the market in 2005 [74]. Since then, these nanoparticles have been used in a variety of cosmetic formulations, mainly in restructuring antiaging creams (e.g., Nanorepair Q10 cream and serum and Nanovital Q10 cream, Dr. Rimpler GmbH) or moisturizing products (e.g., Surmer masque crème hydratante, Surmer crème legère nanoprotection, Isabelle Lancray). NLC concentrates such as NanoLipid Restore CLR™ (Ribes Nigrum (Black Currant) Seed Oil (and) Copernicia Cerifera (Carnauba) Wax (and) Decyl Glucoside (and) Water) are also available and facilitate the development of tailormade formulations according to cosmetic companies’ strategies [39].

Even if it is estimated that more than 500 cosmetic products containing NLCs are available, these products are not explicitly listed as NLCs in the INCI nomenclature, only the individual components of NLCs, such as actives, lipids, and surfactants [39].

## 7. Recent Patents and Patent Applications with SLNs and NLCs

The advantages offered by SLNs and NLCs in applications for the cosmetic industry have been intensively investigated, and numerous patent applications on this topic have been filed in the last years. Some recently filed patents, summarized in Table 4, highlight the potential applications of SLNs and NLCs in cosmetic and therapeutical fields. These patents emphasize the abovementioned advantages in the previous subsections, mainly the efficient delivery of active ingredients, enhanced bioavailability, and improved stability in various formulations.

Patents related to cosmetic products for face care include skin whitening, antiaging, moisturizing, and antiacne formulations, and incorporate different actives such as phenethyl resorcinol, ceramides, and azelaic acid. The preparation of SLNs and NLCs with PCs such as curcumin, astaxanthin, glabridin, and arbutin has been addressed in a few patents [75,76]. The incorporation of antioxidants such as curcumin ensures a good entrapment efficacy and superior solubilization (10,000 times higher than the free active) through an easy scalable preparation method with potential industrial application [77]. For another powerful antioxidant molecule, astaxanthin, an improved solubility and better stability were obtained when this phytocompound was entrapped in SLNs, with a mass ratio of the lipid material and water of 1:11, a lipid phase consisting of solid lipid 1–15%, vegetable oil 85–98.9%, and astaxanthin, 0.1%; in the aqueous phase, a ratio of 1–10% surfactant was used. The entrapment of essential oils in solid-lipid nanostructures is particularly advantageous for improving the physical and chemical stability of the phytocomplex. Thus, in the case of α-tocopheryl stearate-SLNs loaded with bergamot oil, stability studies showed that after two and six months of light exposure, the level of active ingredients decreased by only 10% and 18%, respectively, and no further degradation was observed at twelve months [78].

The use of plant extracts in SLNs and NLCs has potential applications that can be valorized into patent applications related to unique combinations of plant extracts with synergistic effects. Thus, patent CN116211777A describes preparation of SLNs with concentrated extracts of *Angelica dahurica*, *Scutellaria baicalensis*, and *Forsythia chinensis* and, further, the incorporation in a semisolid vehicle. The product displayed in vitro antioxidant, tyrosinase inhibitory activity, and antibacterial activity on *Cutibacterium acnes* strains [79].

Patents related to body care products employ NLCs in noninvasive approaches targeting fat deposits, emphasizing their potential in body contouring and lipolysis treatments [80]. In sunscreen formulations, the sunscreen filters encapsulated NLCs demonstrated an increase in the photoprotection effect [81].

Patents related to hair care products describe SLNs and NLCs to improve hair fiber properties, emphasizing the suitability of NLCs and SLNs as vehicles for releasing active ingredients on hair and scalp surfaces [82].

The abovementioned patents describe several innovative products and preparation methods and show the advancements achieved within the lipid nanoparticles field. Recent applications describe specialized formulations such as pH-responsive or mucoadhesive SLNs, offering solutions for an efficient delivery of active substances [83,84].

**Table 4:** Selection of recent patents and patent applications with SLNs and NLCs.

No	Patent No	Title	Filing Date	Description	Ref.
1	CN104257632A	Solid lipid nanometer particle for astaxanthin and preparation method of solid lipid nanometer particle.	2014-10-24	Astaxanthin SLNs.	[76]
2	CA2872279A1	Topical lipolysis compositions and methods.	2014-11-25	NLCs targeting fat deposits for noninvasive therapy of aggregated deposits of adipose tissue.	[80]
3	KR101860555B1	Solid lipid nanoparticles composition for skin-whitening effect comprising MHY498 and preparation method thereof.	2016-09-12	Skin-whitening SLNs composed of a core layer made of MHY498 and a lipid matrix, and a shell layer made off Poloxamer 188 as an active ingredient.	[85]
4	JP7010823B2	Compounds useful in the treatment and/or care of skin, hair, nails and / or mucous membranes.	2016-12-08	SLNs and NLCs incorporated in cosmetic delivery systems for antiaging properties.	[86]
5	US2021069121A1	Solid lipid nanoparticle for intracellular release of active substances and method for production the same.	2017-12-12	SLNs containing a lipid (natural plant or synthetic wax), a surface acting agent (d-α-Tocopheryl polyethylene glycol 1000 succinate), water and an active compound.	[87]
6	US2021353553A1	Mucoadhesive dispersion nanoparticle system and method for production the same.	2018-09-11	Mucoadhesive dispersion nanoparticle system for the transport and delivery of actives entrapped in SLNs.	[84]
7	US2022151945A1	Solid lipid nanoparticles of curcumin.	2018-11-26	Curcumin-loaded SLNs using generally recognized as safe (GRAS) components.	[77]
8	KR20200094871A	Lipid-protein nanocomposites comprising ginsenoside and use thereof.	2019-01-30	Lipid nanocomplex containing ginsenoside, phosphatidylcholine, sucrose fatty acid ester, surfactant, and positively charged protein.	[88]
9	JP7377813B2	Hair modification composition and method thereof.	2019-04-11	Hair care products with SLNs and NLCs.	[82]
10	CN111988999A	Pigment-loaded solid lipid nanoparticles.	2019-04-16	SLNs containing oil-soluble pigments and high melting point lipids in the core and a surfactant system formed by polysorbate and phospholipids.	[89]
11	CN112691044A	Positive charge modified solid lipid nanoparticle and preparation method thereof.	2019-10-22	SLNs for transdermal administration of cosmetic actives.	[90]
12	CN110974712A	Whitening acid nanostructure lipid carrier as well as preparation method and application thereof.	2019-12-27	Whitening acid nanostructured lipid carrier including the following components: fat-soluble acid, emulsifier, water-soluble acid, emulsion stabilizer, water.	[91]
13	US2022265565A1	Nanotechnology-based delivery system of bergamot essential oil, method of preparation of the system and uses thereof.	2020-07-29	α-tocopheryl stearate-solid lipid nanostructures loaded with bergamot essential oil without psoralens.	[78]
14	CN114515258A	Phenylethyl resorcinol nanostructure lipid carrier, preparation method, and application in cosmetics.	2020-11-20	Phenethyl resorcinol nanostructure lipid carrier for cosmetic applications.	[92]
15	KR102577778B1	pH-responsive capsosome using chitosan-coated solid lipid nanoparticles as core.	2020-12-24	pH-responsive-release-controlled capsosome prepared by reacting liposomes with chitosan-coated SLNs.	[83]
16	EP4169509A1	Particle containing lipid nanoparticles and method for producing same.	2021-05-18	SLNs included in functional cosmetics.	[93]
17	WO2022112527A1	Method for preparing nanosystems.	2021-11-26	Lipid-based nanoparticulate carriers, preferably SLNs or NLCs that be entrapped in vehicles for cosmetic use.	[94]
18	CN112868816A	Preparation method of water-in-oil emulsion gel based on diglyceride solid lipid nanoparticles.	2021-01-26	W/O emulsion gel based on diglyceride SLNs.	[95]
19	TWM639079U	Mist spray container bottle with nanostructured lipid carrier suspension.	2021-09-29	Spray container bottle with NLC suspension.	[96]
20	CN113876642A	Face cream composition containing lipid nanoparticles with moisturizing and hydrating effects and preparation method thereof.	2021-11-12	Face cream composition containing lipid nanoparticles with moisturizing effect.	[97]
21	KR20230149537A	Solid lipid nanoparticles comprising cosmetic composition and cosmetics comprising the same.	2022-04-20	SLNs containing 4-alkylresorcinol and cosmetic compositions containing SLNs with 4-alkylresorcinol.	[98]
22	WO2023137532A1	Nanostructured lipid carrier, use of the nanostructured lipid carrier, photoprotective composition and method for skin photoprotection.	2022-01-19	NLCs consisting of at least one solid natural lipid, at least one liquid natural lipid, and at least one surfactant, which are combined to encapsulate sunscreen filters and enhance photoprotection in a sunscreen formulation.	[81]
23	CN116139025A	Preparation and application of multi-repeat-matching nanostructure lipid carrier.	2022-08-26	Multiple complex type NLC with glabridin and arbutin.	[75]
24	CN115517988A	Azelaic acid nano lipid particle, freeze-dried powder, preparation method and application.	2022-10-12	Nanolipid particles containing azelaic acid, cetyl palmitate, oleic acid, emulsifier (Tween 80 or poloxamer), glycerin, and water.	[99]
25	CN116211777A	Whitening and acne-removing cosmetic and preparation process thereof.	2023-04-24	SLNs with concentrated extracts of Angelica dahurica, Scutellaria baicalensis, and Forsythia chinensis.	[79]
26	CN116327662A	Antiaging solid lipid nanoparticle emulsion and preparation method thereof.	2023-05-06	Antiaging SLN emulsion containing 3,3,5-trimethylcyclohexyl dimethylamide succinate.	[100]
27	CN116725918A	Preparation method and application of diglyceride nanostructure lipid carrier hydrogel.	2023-05-31	Diglyceride caprylic acid NLC and diglyceride caprylic acid NLC hydrogel containing vegetable oil, diglyceride, soybean lecithin, tea saponin and glycerin.	[101]
28	CN117137825A	Ceramide lipid nanoparticles as well as preparation method and application thereof.	2023-09-07	Ceramide lipid nanoparticles.	[102]
29	CN117398294A	Chitosan interface modified ellagic acid nanostructure lipid carrier and preparation method thereof.	2023-10-16	NLCs loaded containing ellagic acid, solid lipids, liquid lipids, and emulsifier sodium caseinate.	[103]
30	WO2024102798A1	Lipid nanoparticles as active molecule carriers in ophthalmic, dermatological, and/or cosmetic applications, and process for production thereof.	2023-11-08	NLCs containing solid lipids (glyceryl distearate, cocoa butter, and combinations thereof), liquid lipids (Linoleoyl Polyoxyl-6 glycerides, Oleoyl Polyoxyl-6 glycerides and combinations thereof) and nonionic surfactants (Polyethylene glycol 660 12-hydoxystearate, Polysorbate 80, and combinations thereof).	[104]

## 8. Conclusions

There is growing interest in SLNs and NLCs development, to explore their potential for topical applications such as cosmetics or dermatological systems, being recognized as efficient carriers and having advantages for large-scale industrial production. They offer significant benefits in enhancing the stability and bioavailability of natural bioactives due to their protection and improved stability. Moreover, SLNs and NLCs may enhance the delivery and penetration of HEs, EOs, oils, and PCs, ensuring targeted and controlled release. The film formation and adherence on the skin of the SLNs and NLCs can increase the hydration and protection of the skin and, consequently, the penetration of natural bioactives. All of these can enhance the effects of bioactives for skin health for the benefit of patients or consumers. Recent research and patent applications in the field of SLNs and NLCs loading natural bioactives for skin health have shown significant advancements in encapsulation efficiency, stability, and bioactives delivery. The choice of lipids, surfactants, and preparation methods critically influenced the physicochemical properties and efficacy of the final formulations. In vitro and in vivo studies evaluated the potential of these nanoparticles to enhance the therapeutic outcomes of natural bioactives, providing promising opportunities for advanced skincare and dermatological treatments. Further research could focus on optimizing formulations for specific conditions, improving efficacy, and conducting extensive in vivo studies to validate these findings. However, their full potential has not been fully explored yet, while new natural bioactives can be encapsulated in these nanosystems; consequently, this research field will be continuously growing to create more appealing products.

## Figures and Tables

**Table 1:** The main SLNs and NLCs categories and their key features.

SLNs and NLCs Categories		Characteristics
**SLNs**	Type I (homogenous matrix model)	The bioactive is dispersed in the lipid core.
	Type II (drug-enriched shell model)	The lipid core is bioactive-free, and the exterior solid shell has both lipids and bioactive.
	Type III (drug-enriched core model)	The active ingredient is precipitated in the core with a lipid coverage (the active concentration is close to its saturation solubility in the lipid).
**NLCs**	Type I (imperfect crystal model)	The mixture of lipids has a great number of voids and imperfections where the bioactive can be placed.
	Type II (amorphous/structureless model)	The special lipids do not recrystallize after homogenization and cooling and an amorphous lipid matrix that minimizes drug expulsion.
	Type III (multiple model)	Small oil nanoparticles that are inside the solid lipid matrix due to a phase separation (mixing solid lipids with higher amounts of oils in a ratio where the solubility of the oil in the solid lipid is exceeded).
